# Book Reviews

**Published:** 1907-09

**Authors:** 


### BOOK REVIEWS.

A TEXT-BOOK OF PHARMACOLOGY. New (2d) Edition, Adapted to the 1905 Pharmacopeia. A Text-Book of Pharmacology. Including Therapeutics, Materia Medica, Pharmacy, Prescription-Writing, Toxicology, etc. By Torald Sollmann, M. D., Asst. Professor of Pharmacology and Materia Medica, Western Reserve University, Cleveland, Ohio. New (2d) edition· Octavo of 1070 pages, fully illustrated. Philadelphia and London: W. B. Saunders Company, 1906. Cloth, $4.00 net; half morocco, $5.00 net.

Dr. Sollmann has revised his work to conform with the new (1905) Pharmacopeia. He has based the study of therapeutics on a systematic knowledge of the nature and properties of drugs, thus bringing out forcibly the intimate relation between pharmacology and practical medicine. Speaking of the book, Dr. J. F. Fotheringham, Professor of Therapeutics and Theory and Practice of Prescribing, Trinity Medical College, Toronto, says: "The work certainly occupies ground not covered in so concise, useful, and scientific a manner by any other text I have read on the subjects embraced." The British Medical Journal finds the book "well worthy the attention of all who are interested in the pharmacologic and physiologic side of therapeutics." The work has been entirely reset.

MATERIA MEDICA AND THERAPEUTICS. By Thos. S. Blair, M. D. The Medical Council, Philadelphia.

Physicians, without respect to school affiliations, will find this little book of benefit and use to them. It is very practical, largely founded on the author's personal experience and is not confined to drugs contained in the United States Dispensary and the National Formulary· The best of everything seems to be the author's conception of a book of materia medica and therapeutics, and this he has given.

THE PRACTICE OF GYNECOLOGY.—New (3d) Edition, Thoroughly Revised. A Text-Book on the Practice of Gynecology. For Practitioners and Students. By W. Easterly Ashton, M. D., LL.D., Professor of Gynecology in the Medico-Chirurgical College of Philadelphia. Third Edition, Thoroughly Revised. Octavo of 1096 pages, with 1057 original line drawings. Philadelphia and London: W. B. Saunders Company, 1906. Cloth, $6.50 net; half morocco, $7.50 net.

Three editions of Dr. Ashton's excellent work have been required in eighteen months, demonstrating that the medical profession were quick to appreciate the practical value of the work. He not only tells what should be done, but also precisely how to do it. The 1057 drawings show in detail the procedures and operations without obscuring their purpose by unnecessary anatomic surroundings.

---

MATERIA MEDICA FOR NURSES· Including Therapeutics and Toxicology. A Text-Book of Materia Medica for Nurses. Including Therapeutics and Toxicology. By George P. Paul, M. D., Assistant Visiting Physician and Adjunct Radiographer to the Samaritan Hospital, Troy, N. Y., 12 mo of 240 pages. Philadelphia and London: W. B. Saunders Company, 1907. Cloth, $1.50 net.

In Dr. Paul's new work the drugs themselves are arranged alphabetically, and their physiologic actions according to the action of the drug and not the organ acted upon; thus, with a glance, the full actions of any drug may be seen. Another important section is that on pretoxic signs, giving the warnings of the full action or the beginning toxic effects of the drug, if heeded, may prevent many cases of drug poisoning. The nurse should know these signs. The book will form a valuable part of the nurse's equipment.

---

At the annual meeting of the American Medical Editor's Association, held in Atlantic City last month, the following officers were elected: Charles S. Taylor, Philadelphia, Medical World, president; Kenneth W. Millican, St. Louis, St. Louis, Medical Review, first vice-president; H. Edwin Lewis, New York City, International Journal of Surgery, second vice-president; Joseph McDonald, Jr., New York City, American Journal of Surgery, secretary-treasurer. Wallace C. Abbot, Chicago; William A. Young, Toronto, and David C. English, New Brunswich, New Jersey, executive council.

